# Highly Potent Neutralizing Nanobodies Acting Against Chikungunya Virus Infection via Inhibiting Multiple Stages of the Viral Life Cycle

**DOI:** 10.3390/ijms26093982

**Published:** 2025-04-23

**Authors:** Liyuan Song, Guangcheng Fu, Jie Li, Zhengshan Chen, Ling Fu, Changming Yu, Li Qiang, Jiangfan Li, Ting Fang, Hongyu Yuan, Jianmin Li

**Affiliations:** 1 Laboratory of Advanced Biotechnology, Beijing Institute of Biotechnology, Beijing 100071, China; 2 Laboratory of Advanced Biotechnology, ZJU-Hangzhou Global Scientific and Technological Innovation Center, Hangzhou 311215, China; 3 College of Pharmacy, Nanjing University of Chinese Medicine, Nanjing 210023, China

**Keywords:** Chikungunya virus, p62-E1 heterodimer, alpaca, nanobody, neutralizing antibody

## Abstract

The Chikungunya virus (CHIKV) is a priority endemic pathogen identified by the World Health Organization and its infection induces an acute febrile illness in humans that is often associated with arthritis and musculoskeletal pain. Therefore, specific vaccines and treatments are urgently needed to prevent or treat Chikungunya disease. Here, we identify a series of CHIKV-specific neutralizing nanobodies (Nbs) from an alpaca which exhibit distinct binding modes compared to those previously reported. Two representative anti-CHIKV Nbs, N033-Fc and N053-Fc, demonstrated significant antiviral activity in *Ifnar^−/−^* mice against lethal challenge. Further studies elucidated the functional mechanisms of N033-Fc and N053-Fc in blocking CHIKV infection at multiple stages of the viral life cycle. This study identifies multiple candidate Nbs that may be suitable for next-generation antibody therapies to combat CHIKV infection.

## 1. Introduction

The acceleration of globalization and the dynamic spread of mosquitoes between endemic and non-endemic regions have led to the expansion of mosquito-borne diseases, posing a significant threat to public health [1]. The Chikungunya virus (CHIKV) is a typical widespread Aedes mosquito-borne alphavirus that imposes a considerable economic burden in affected areas [2]. CHIKV infection has been associated with a spectrum of acute or chronic clinical symptoms including joint swelling, muscle pain, headache, and rash, which significantly impact patients’ quality of life [3].

Over the past 20 years, more than 10 million cases of CHIKV infection have been reported in approximately 110 countries and territories [4,5,6], and the rapid spread of CHIKV has made it necessary to develop prophylactic strategies or effective antiviral treatments to control the virus. The single-dose live-attenuated vaccine Ixchiq (VLA1553) was approved by the Food and Drug Administration (FDA) in 2023, and phase 3 clinical trials demonstrated its overall safety and efficacy against CHIKV infection [7,8,9]. Another effective vaccine is the virus-like particle (VLP) vaccine, which was designed based on the structural components of CHIKV and has shown clinical safety while eliciting durable immune responses [10]. Other CHIKV vaccine platforms, such as mRNA vaccines and virus-vector-based vaccines, are currently undergoing preclinical studies [11]. So far, monoclonal antibody (mAb)-based therapies have made significant progress in protecting animals from alphavirus infection [12]. Despite the promising progress of antiviral strategies in preclinical models, no CHIKV-specific therapies have been licensed for therapeutic or preventive use in humans. The CHIKV genome encodes five structural proteins (capsid, E3, E2, 6K, and E1), and the virion surface is composed of 80 trimeric E2-E1 heterodimers. Since E2 is primarily responsible for viral attachment and the class II fusion protein E1 regulates viral membrane fusion, protective antibodies mainly target E2 and E1 [13].

Except for traditional forms of mAbs, llama-derived single-domain antibodies, also known as nanobodies, were first reported by Belgian scientists in 1993 [14]. Several properties make nanobodies (Nbs) attractive for therapeutic applications compared to conventional mAbs [15]. First, as many epitopes are hidden in grooves or cavities that are difficult to access, the unique small size of Nbs enables them to identify cryptic epitopes inaccessible to canonical antibodies [16]. Second, nanobodies also exhibit low immunogenicity due to their small size, making them suitable for therapeutic applications. Furthermore, a conserved disulfide bond enhances the stability of VHH under harsh conditions, such as extreme pH and high temperatures [17]. Notably, VHHs possess a longer CDR3 sequence, enabling more stable and robust antigen recognition and resulting in diverse interaction modes [18]. The unique properties of VHH and its recombinant fragments offer numerous advantages that cannot be replicated by traditional antibodies, positioning them as a powerful tool in antiviral drug development [19].

In this study, we identified a total of 46 Nbs capable of binding to the CHIKV p62-E1 heterodimer from a phage display library. Among these, we discovered 11 Nbs with potent activity against a live-attenuated strain of CHIKV, targeting distinct epitopes, with IC_50_ values ranging from 0.08 to 0.95 μg/mL. Two representative Nbs, N033-Fc and N053-Fc, protected *Ifnar^−/^^−^* mice from lethal challenge in both prophylactic and therapeutic groups. Neutralization mechanism studies revealed that N033-Fc and N053-Fc blocked multiple stages in the viral life cycle, including viral entry, membrane fusion, and viral egress. The unique mechanism of action of these anti-CHIKV Nbs makes them promising drug candidates for future clinical applications.

## 2. Results

### 2.1. Screening and Identification of Nbs Targeting CHIKV p62-E1 Heterodimer

**Figure 1:**
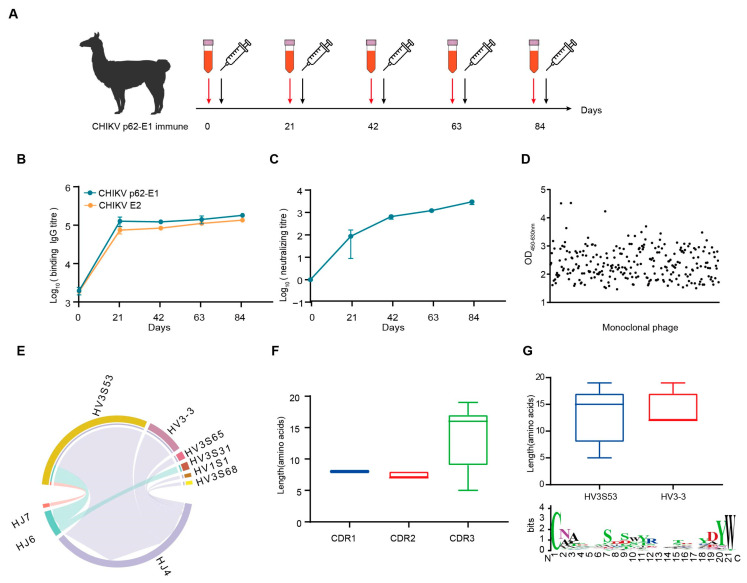
CHIKV-specific Nbs was isolated from an immunized alpaca. (**A**) Timeline of immunization and serum collection. (**B**) The binding titer of the serum to CHIKV p62-E1 and CHIKV E2 at each time point was tested by ELISA. (Data are the mean ± S.D. of three replicates from one representative experiment.) (**C**) The neutralization titers of the serum against CHIKV live-attenuated strain infection. Each dot represents the mean reciprocal of serum dilution at half inhibition. (Data are the mean ± S.D. of three replicates from one representative experiment.) (**D**) Monoclonal phages binding to CHIKV p62-E1 were detected by ELISA. Binding monoclonal phages were defined as OD_450-630_ > 1. (**E**) VJ gene pairwise preference of 53 specific Nbs. (**F**) CDR1, CDR2, and CDR3 amino acid length distribution of 53 specific Nbs. (**G**) CDR3 amino acid length distribution for Nbs derived from either HV3S53 or HV3-3 germlines. A Weblog plot for CDR3 amino acid entropy is shown. The *y*-axis represents the entropy of amino acids, and the *x*-axis represents the position in the variable domain. The larger the letter of the amino acid, the greater the entropy value.

Considering the trimeric feature of CHIKV glycoproteins, we separated the trimeric and monomeric forms of CHIKV p62-E1 by molecular exclusion chromatography (Supplementary Figure S1a,b). To obtain specific Nbs against CHIKV, we sequentially immunized an alpaca with recombinant CHIKV p62-E1 proteins, and screened Nbs by in vitro phage display technology [20]. First, to enhance the immune response and determine the optimum time point for PBMC isolation, an alpaca was immunized with recombinant CHIKV p62-E1 proteins at three-week intervals (Figure 1A). Correspondingly, sera were collected at different time points to detect binding and neutralization antibody titers for CHIKV. The results showed that antibody titers reached 105 for CHIKV p62-E1 and E2 binding, respectively (Figure 1B), and serum neutralization titers against CHIKV infection reached 103 at 84 days post-immunization (Figure 1C). To identify Nbs that specifically bind to CHIKV, supernatants of randomly selected phages were analyzed by ELISA, and, finally, 309 Nbs targeting CHIKV p62-E1 heterodimer were identified (OD_450-630_ > 1) (Figure 1D). Comparative analysis of CDR3 regions excluded Nbs with similar features, and 53 Nbs were selected for further study. To characterize the genetic features of the 53 Nbs, coding sequence analysis was conducted by using the IMGT/V-QUEST program (http://www.imgt.org/IMGT_vquest/vquest/, accessed on 10 January 2025). The frequency and genetic characterization of V(D)J sequences were consistent with those of previous studies in alpacas [21]. A large portion of antibodies utilized the HV3S53 (69.8%, 37/53), HV3-3 (18.9%, 10/53), and HJ4 (84.9%, 45/53) germline genes (Figure 1E). Length distribution analysis of the CDR regions indicated that the CDR3 regions mostly consisted of 17 amino acids, consistent with the typical feature of Nbs (Figure 1F). Analyzing CDR3, we further found that IGHV3S53-derived antibodies have a longer CDR-H3 loop (mean length 14 amino acids) than IGHV3-3-derived antibodies (mean length 12 amino acids). A thorough sequence analysis of the VHH CDR3 region unveiled the diversity of CDR3, which satisfies its intricate requirements in antigen binding processes (Figure 1G). Finally, sequences encoding representative Nbs were assembled into linear cassettes containing a human IgG1 constant region for rapid transient expression in Expi293F cells, termed VHH-Fc. The results showed that 46 Nbs were successfully expressed (Supplementary Figure S2).

### 2.2. Properties of Neutralizing Nbs Against CHIKV

**Figure 2:**
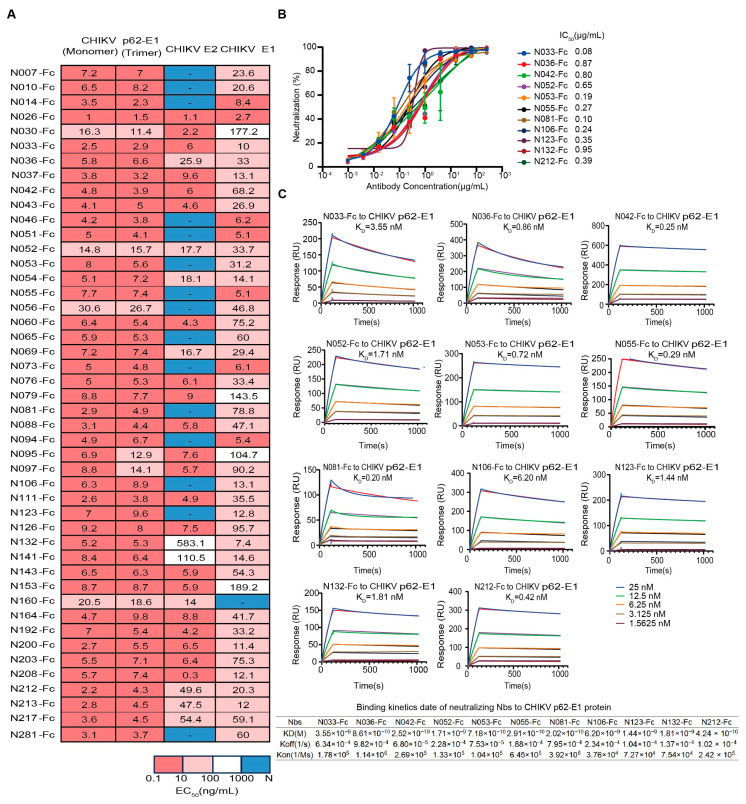
Characterization of isolated CHIKV-specific Nbs. (**A**) Heatmap showing the binding activity (EC_50_) of 46 Nbs selected for further characterization. Deep pink represents 0.1 ng/mL < EC_50_ < 10 ng/mL, light pink represents 10 ng/mL < EC_50_ < 100 ng/mL, white represents 100 ng/mL < EC_50_ < 1000 ng/mL, and navy blue represents EC_50_ > 1000 ng/mL. (Data are representative of two experiments, and the EC_50_ values of each Nb were calculated using GraphPad Prism V8 by performing a 4-parameter non-linear regression dose–response analysis). (**B**) Neutralizing activity of isolated Nbs against a live-attenuated strain of CHIKV. (Data are representative of two experiments and the IC_50_ values of each Nb were calculated using GraphPad Prism V8 by performing a 3-parameter non-linear regression dose–response analysis.) (**C**) SPR sensorgrams showing the binding kinetics of neutralizing Nbs to CHIKV p62-E1. The table below shows the relevant parameters.

First, we assessed the binding characteristics of the 46 expressed Nbs, and most Nbs could simultaneously bind to trimeric and monomeric forms of CHIKV glycoproteins (Figure 2A). We further identified their binding activity to the recombinant CHIKV E2 and E1 proteins. The epitopes of 26 Nbs (Nbs against CHIKV E2 and E1 proteins with EC50 < 100 ng/mL simultaneously) spanned both E2 and E1 (Figure 2A). It is worth mentioning that some Nbs only bound to E2 or E1, implying different recognition mechanisms (Figure 2A). To assess the inhibitory potential of Nbs against the attenuated CHIKV vaccine, focus reduction neutralization tests (FRNTs) were performed on BHK-21 cells. Finally, 11 Nbs that exhibited strong neutralizing activities were identified, with IC50 values ranging from 0.08 μg/mL to 0.95 μg/mL, including five Nbs that could bind both E2 and E1 and other Nbs that bind only E1 (Figure 2A,B). Further studies were performed to measure the binding affinity of neutralizing Nbs to CHIKV p62-E1 proteins by surface plasmon resonance (SPR). Subnanomolar dissociation constants (Kᴅ) were observed for antigen–antibody interactions using a 1:1 kinetic model. The KD values of tested Nbs for CHIKV p62-E1 proteins ranged from subnanomolar to nanomolar affinity (0.2–6 nM) (Figure 2C). These findings were consistent with the ELISA results (Figure 2A).

### 2.3. Neutralizing Nbs Target Different Epitopes on CHIKV p62-E1 Heterodimer

**Figure 3:**
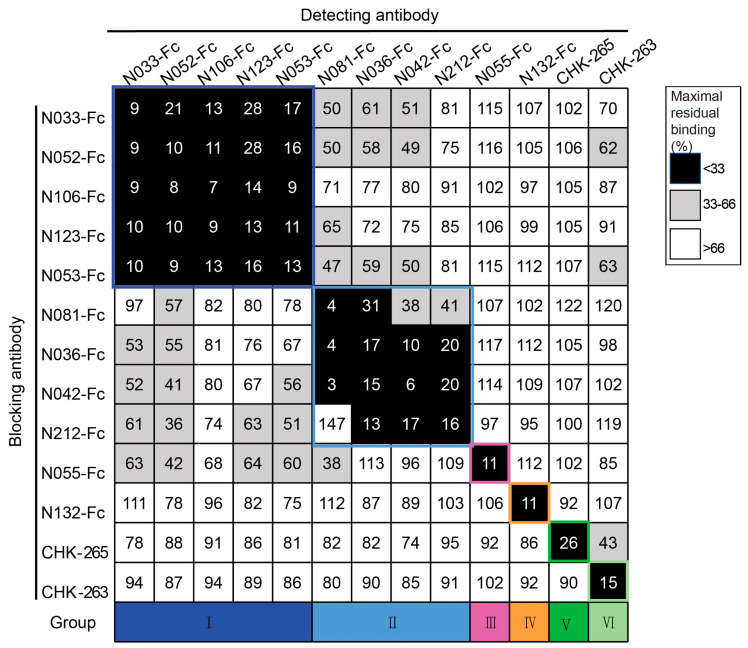
Competition-binding groups of neutralizing Nbs as determined by ELISA using CHIKV p62-E1. Results for a total of 13 Abs are shown (among them, CHK-265 and CHK-263 are the previously reported monoclonal antibodies). The first mAb (20 μg/mL) incubated with CHIKV p62-E1 is shown in the left-hand column, and the second mAb (biotinylated; 1 μg/mL) is shown in the top row. Black boxes indicate competition (reduction in maximal binding to <33%), grey boxes indicate intermediate competition (33% to 66% maximal residual binding), and white boxes indicate no competition (>66% maximal residual binding). Competition-binding groups are indicated by the respective colored boxes. Navy blue boxes indicate group I, light blue boxes indicate group II, purple boxes indicate group III, orange boxes indicate group IV, dark green boxes indicate group V, and pale green boxes indicate group VI. (Data are representative values of 3 independent experiments.).

To classify the antigenic landscape of the 11 neutralizing Nbs, a competition ELISA was conducted to clarify their binding modes compared to antibodies with defined epitopes. Unbiotinylated Nbs were incubated with CHIKV p62-E1 proteins first, and then biotinylated Nbs were added for detection. A decrease in the relative binding signal of the biotinylated Nbs implied competitive binding with the unbiotinylated Nbs. Finally, the binding modes of neutralizing Nbs were classified into four groups: group I (N033-Fc, N052-Fc, N106-Fc, N123-Fc, and N053-Fc), group II (N081-Fc, N036-Fc, N042-Fc, and N212-Fc), group III (N055-Fc), and group IV (N132-Fc). Meanwhile, we found that they did not compete with the previously reported antibodies CHK-265 [22] (E2 B domain) and CHK-263 [23] (E1 and E2) (Figure 3), implying a distinct recognition pattern. Since multiple Nbs showed neutralizing activity and formed unique competition groups, we selected N033-Fc and N053-Fc, which had the lowest IC_50_ of group I, for protective activity evaluation in vivo.

### 2.4. Prophylaxis and Therapeutic Efficacy of N033-Fc and N053-Fc In Vivo

For efficacy evaluation of the N033-Fc and N053-Fc inhibitory activity in vivo, a lethal mouse model of CHIKV was established in adult mice lacking the type I interferon receptor (Ifnar^−/−^) and C57BL/6 mice by subcutaneous inoculation in the foot [24]. Infection of Ifnar^−/−^ C57BL/6 mice with an attenuated CHIKV vaccine of 1 × 10^5^, 1 × 10^4^, and 1 × 10^3^ TCID_50_ and signs of morbidity or mortality were observed at 48, 60, and 72 h post-infection. As a result, infected mice were dead at 72 h post-infection (h) and a lethal model was established with a challenge titer of 1 × 10^3^ TCID_50_ (Supplementary Figure S3a,b).

**Figure 4:**
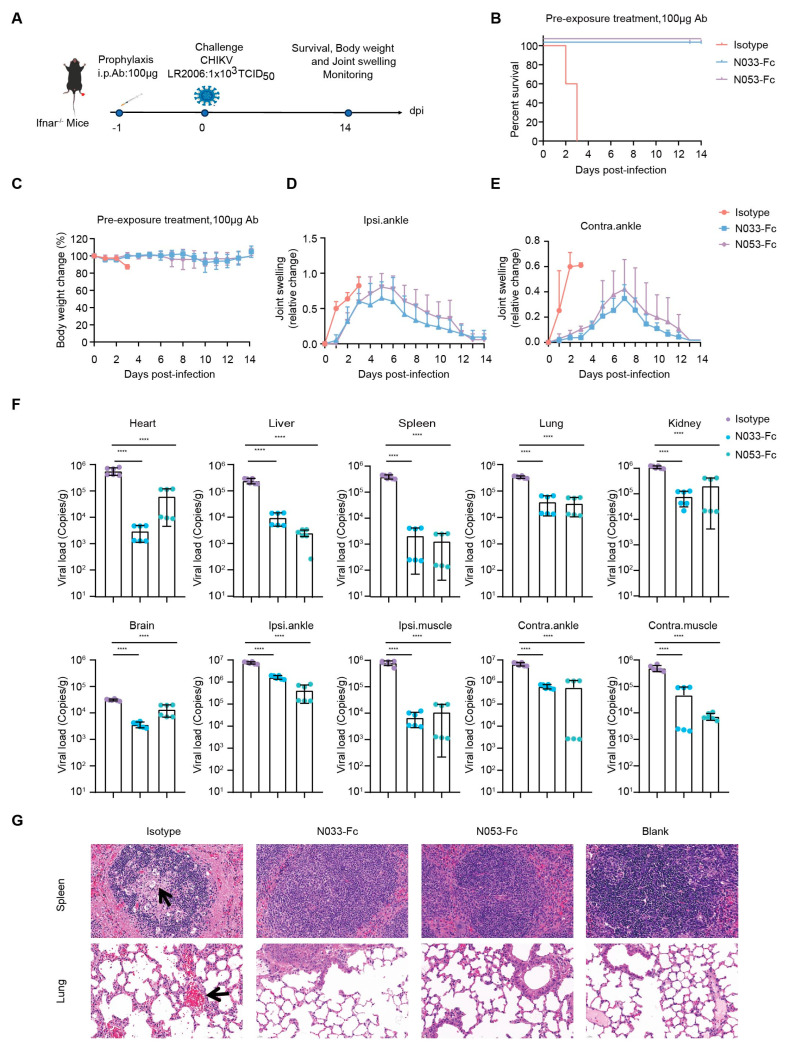
Efficacy of the prophylaxis of two representative neutralizing Nbs, N033-Fc and N053-Fc. Four-to-six-week-old *Ifnar^−/^^−^* mice were treated with 100 μg of N033-Fc, N053-Fc, and isotype antibody RVFV-NA137-Fc 1 day before subcutaneous inoculation with a live-attenuated strain of CHIKV. (**A**) Experimental schedule for Nb prophylaxis. (**B**) Survival (survival curve analysis was performed using the log-rank test). (**C**) Weight change, (**D**) ipsilateral foot swelling, and (**E**) contralateral foot swelling were monitored and scored for 14 days after infection. (**F**) Virological assessment by RT-qPCR at 3 dpi in the heart, liver, spleen, lung, kidney, brain, ipsilateral ankle/muscle, and contralateral ankle/muscle. Significance was determined by one-way ANOVA with Dunnett’s post hoc test compared to the isotype control (**** *p* < 0.0001). (**G**) Histological analysis of tissues in mice treated with the indicated Nbs. Black arrows indicate areas of inflammatory cell infiltrates. Representative images of 3 individual mice per group are shown. All images have the same magnification. Scale bar, 200 μm.

To assess the prophylaxis efficacy, a single 100 μg dose of N033-Fc or N053-Fc was administered by intraperitoneal (i.p.) injection 24 h prior to subcutaneous inoculation (s.c.) in the left rear footpad of Ifnar^−/−^ mice challenged with the attenuated CHIKV vaccine of 1 × 10^3^ TCID_50_ (Figure 4A). Survival rates, body weight, and joint swelling symptoms were monitored and recorded for 14 dpi (Figure 4B–E). As predicted, Ifnar^−/−^ mice died 3 dpi when treated with an isotype antibody (Figure 4B). Treatment with N033-Fc and N053-Fc prior to CHIKV inoculation resulted in a 100% survival rate and significantly prevented weight loss (Figure 4B,C). In addition, the prophylactic treatment group exhibited effectively reduced ipsilateral and contralateral foot swelling symptoms post-infection (Supplementary Figure S4, Figure 4D,E). This phenomenon was consistent with the reduced levels of viral RNA in the infected tissues and ankles 3 dpi compared to the isotype control group (Figure 4F). Similarly, among the N033-Fc and N053-Fc treatment groups, viral loads in heart, liver, spleen, lung, kidney, brain, ipsilateral muscle/ankle, and contralateral muscle/ankle were significantly lower than those of the isotype control group (Figure 4F). Analysis of pathological tissues 3 dpi showed that spleen and lung tissues in the N033-Fc- and N053-Fc-administered groups remained intact, while the isotype control group showed inflammatory infiltration (Figure 4G).

**Figure 5:**
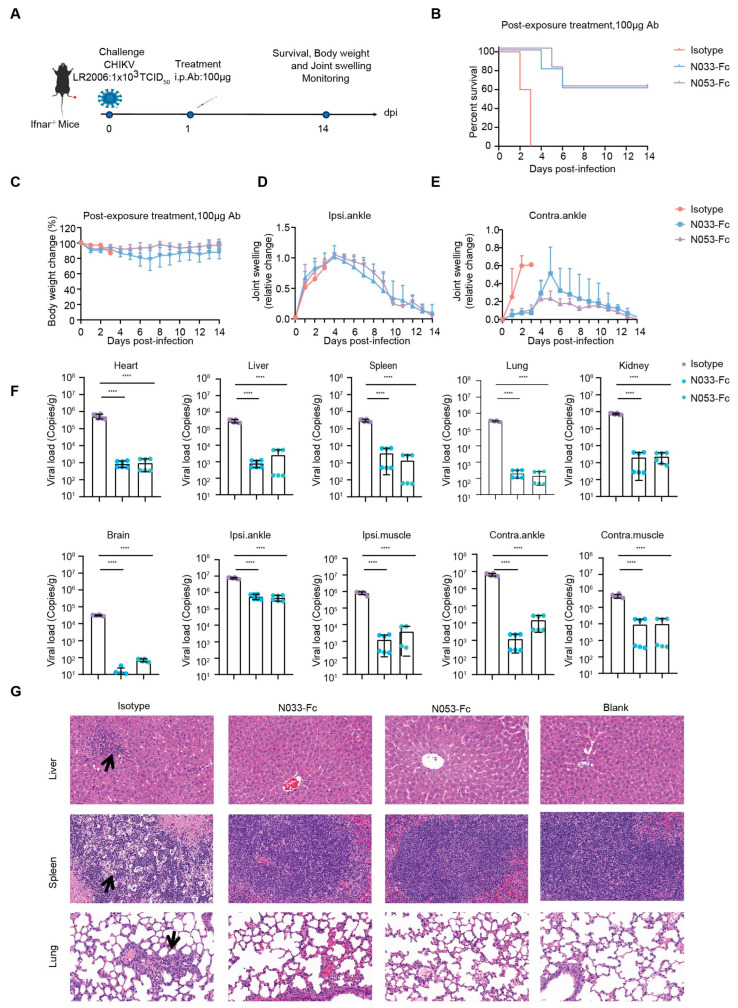
Efficacy of therapy using two representative anti-CHIKV Nbs, N033-Fc and N053-Fc. Four-to-six-week-old Ifnar^−/−^ mice were treated with 100 μg of N033-Fc, N053-Fc, and isotype antibody RVFV-NA137-Fc 1 day after subcutaneous inoculation with a live-attenuated strain of CHIKV. (**A**) Experimental schedule for Nb therapy. (**B**) Survival (survival curve analysis was performed using the log-rank test). (**C**) Weight change, (**D**) ipsilateral foot swelling, and (**E**) contralateral foot swelling were monitored and scored for 14 days after infection. (**F**) Virological assessment by qPCR at 3 dpi in the heart, liver, spleen, lung, kidney, brain, ipsilateral ankle/muscle, and contralateral ankle/muscle. Significance was determined by one-way ANOVA with Dunnett’s post hoc test compared to the isotype control (**** *p* < 0.0001). (**G**) Histological analysis of tissues in mice treated with the indicated Nbs. Black arrows indicate areas of inflammatory cell infiltrates. Representative images of 3 individual mice per group are shown. All images have the same magnification. Scale bar, 200 μm.

To investigate the therapeutic efficacy, a single 100 μg dose of N033-Fc or N053-Fc was administered 24 h post-infection, and, as previously described, survival rates, body weight, and joint swelling symptoms were continuously monitored for 14 days (Figure 5A–E). Animals in the N033-Fc or N053-Fc treatment groups showed a 60% survival rate with minimal loss in body weight (Figure 5B,C). Regarding foot swelling, N033-Fc and N053-Fc effectively relieved the symptoms to nearly baseline levels by 14 dpi (Supplementary Figure S5, Figure 5D,E). For viral titer and pathogenesis analysis, three mice from each group were sacrificed at 3 dpi. Similarly, among the N033-Fc and N053-Fc treatment groups, viral loads in the heart, liver, spleen, lung, kidney, brain, ipsilateral muscle/ankle, and contralateral muscle/ankle were significantly lower than those of the isotype control group (Figure 5F). Histopathology assays indicated that the N033-Fc and N053-Fc treatment groups exhibited effectively reduced liver, spleen, and lung lesion features compared to the control group (Figure 5G).

These results suggest that N033-Fc and N053-Fc effectively limited viral replication and protected Ifnar^−/−^ mice against lethal challenge with the attenuated CHIKV vaccine, highlighting their potent prophylactic and therapeutic efficacy in clinical applications.

### 2.5. N033-Fc and N053-Fc Target Multiple Pathways in the Viral Infection Process

**Figure 6:**
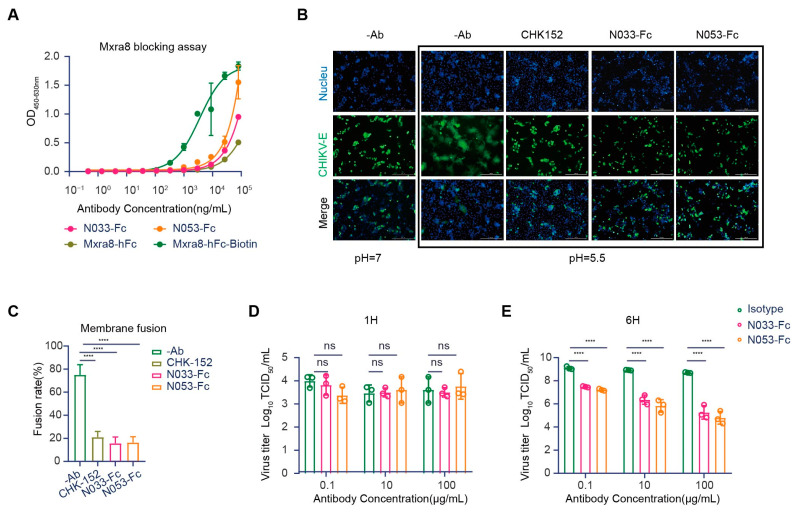
Mechanism of neutralization by anti-CHIKV Nbs. (**A**) Blocking of Mxra8-Fc binding to N033-Fc or N053-Fc complexed with CHIKV was determined by competition ELISA. Data are representative of two experiments, and binding curves were generated by performing a 4-parameter non-linear regression dose–response analysis. (**B**,**C**) N033-Fc or N053-Fc inhibits membrane fusion in a fusion inhibition assay. (CHK-152 is the previously reported monoclonal antibody, as a positive control.) Images of CHIKV cell–cell fusion in the presence of 100 μg/mL N033-Fc or N053-Fc are shown. Scale bars, 50 μm. Analysis of the inhibitory activities of these antibodies against CHIKV cell–cell fusion is presented. The mean ± S.D. from three biologically independent experiments is shown. (**D**,**E**) Egress inhibition studies. Vero cells were inoculated with CHIKV for 1 h, washed extensively to remove unbound virus, and incubated with N033-Fc, N053-Fc, or isotype antibody RVFV-NA137-Fc. Supernatants were harvested at 1 h (**D**) or 6 h (**E**) post-infection, and viral RNA was quantified by RT-qPCR. Data are the mean ± S.D. from three independent experiments. Significance was determined by one-way ANOVA with Dunnett’s post hoc test compared to the isotype control (“ns” not significant; **** *p* < 0.0001).

Evidence has shown that Mxra8 acts as a functional receptor for multiple arthritogenic alphaviruses, including CHIKV, MAYV, ONNV, RRV, and SFV, but not for encephalitic alphaviruses or SINV [25]. Recently, direct binding was observed between Mxra8 and CHIKV, which could be abrogated by anti-CHIKV mAbs [26]. X-ray crystallography and cryo-electron microscopy (cryo-EM) studies confirmed that Mxra8 directly binds across different E1-E2 heterodimers on the virion surface [27]. Accordingly, we first evaluated whether N033-Fc and N053-Fc could block Mxra8 binding to CHIKV using a competition-binding ELISA assay. Compared with the binding curves of human Mxra8-Fc, the results suggested that N033-Fc and N053-Fc blocked Mxra8 binding to CHIKV p62-E1 heterodimer (Figure 6A).

Another mechanism for antibody neutralization of enveloped viruses occurs by inhibiting the membrane fusion process [12]. The CHIKV glycoprotein E1 is a class II fusion protein that comprises three domains (I, II, and III) and mediates viral membrane fusion via a hydrophobic peptide [28]. Although the fusion peptide is normally hidden in E2 domain B, in a low-pH environment, the p62-E1 protein undergoes rearrangement, which enables the exposure of the E1 fusion peptide [29]. To investigate whether N033-Fc and N053-Fc could inhibit the viral fusion process, a cell–cell fusion assay was conducted [30]. As expected, the cell–cell fusion phenomenon was not observed when cells were cultured at neutral pH. Strikingly, N033-Fc and N053-Fc exhibited high potency in inhibiting the cell–cell fusion process at low pH compared with the control group (Figure 6B,C). As CHK-152 was presumed to mediate membrane fusion, it was used as a positive control (Figure 6B,C). These studies indicated that N033-Fc and N053-Fc efficiently inhibited virus fusion with host cell membranes.

Several studies have reported that anti-CHIKV antibodies could inhibit virion release from infected cells [22,31]. We next evaluated whether N033-Fc and N053-Fc could block the viral egress process, presumably by inhibiting the virus assembly or budding from the plasma membrane. Cells were inoculated with the virus and then washed extensively to remove free virus. Then, N033-Fc, N053-Fc, and the isotype control Nb were added, and viral RNA copies of cell supernatants were detected at 1 h or 6 h. As expected, the viral RNA level at 1 h was low, indicating that the washing step removed the unbound inoculum (Figure 6D). At 6 h, we measured the amount of viral RNA. Compared to the isotype control Nb-treated cells, N033-Fc and N053-Fc had a greater ability to inhibit viral egress (Figure 6E). This indicates that newly generated virions were secreted. Furthermore, N033-Fc and N053-Fc reduced the number of CHIKV RNA copies in the supernatants compared to the control group at 6 h. Thus, N033-Fc and N053-Fc possess the ability to block virus egress.

## 3. Discussion

CHIKV is widespread in tropical regions and causes recurrent outbreaks of Chikungunya fever (CHIKF), which is characterized by a self-limiting illness with acute fever, chronic joint pain, and, in some patients, prolonged arthralgia [32]. CHIKF can lead to significant disability and impose a substantial economic burden in endemic regions, with higher mortality rates among vulnerable populations such as infants, the elderly, and individuals with pre-existing medical conditions [33,34,35]. Despite the approval of a live-attenuated vaccine, the potential for a global outbreak of CHIKF in the coming years underscores the need for additional effective treatment options [3].

Nbs are highly versatile due to their small size, tissue penetration, stability, low immunogenicity, and ease of modification [36]. They can target epitopes inaccessible to traditional antibodies and have shown promise in both clinical trials and animal models [37,38]. Nbs are primarily generated via phage display libraries, enabling the rapid production of high-affinity antibodies [25]. However, no anti-CHIKV neutralizing Nbs have been described to date, highlighting a potential area for advancing CHIKV prevention and treatment.

In this study, we genetically engineered VHH antibodies, identified highly neutralizing anti-CHIKV Nbs using the phage display technique, and further investigated their inhibition mechanisms and protective activity in vivo. First, we isolated and characterized single-domain antibodies from an alpaca immunized with recombinant CHIKV p62-E1 proteins. As expected, these VHHs bound to both the monomeric and trimeric antigens with high affinity, which implied that the recombinant CHIKV p62-E1 proteins effectively provoked antigen-specific responses in the alpaca. Second, we evaluated the inhibitory activity of CHIKV-specific Nbs on BHK-21 cells, and 11 neutralizing Nbs were identified. In addition, we analyzed the epitopes of the 11 Nbs through competitive-binding ELISA and classified them into four main groups. Interestingly, the binding motifs of these Nbs spanned both E2 and E1 or specifically bound to E2 or E1, which did not overlap with known protective mAbs, highlighting the unique and diverse recognition modes of Nbs [24,27,39]. Then, we selected nNbs N033-Fc and N053-Fc (from group I), which had the highest potency, for a prophylactic and therapeutic protection study in a mouse model. Significant prophylactic and therapeutic protective efficacy was observed for N033-Fc and N053-Fc when administered 1 day pre-infection or post-infection. In addition, we demonstrated that N033-Fc and N053-Fc could inhibit infection at multiple steps of the viral life cycle, including both the entry and egress processes.

In conclusion, since N033-Fc could simultaneously bind to both E2 and E1, whereas N053-Fc only targeted E1, and they both belonged to group I and competed with each other, we concluded that group I Nbs may recognize a flexible epitope spanning E2 and E1 and are promising drug candidates worthy of further investigation. In addition, therapeutic treatment with N033-Fc and N053-Fc was verified to inhibit the infection of live-attenuated CHIKV in Ifnar^−/−^ mice with a protective efficacy of 60%, whereas the efficacy was 100% in a pre-experimental model. These differences may stem from the following factors: First, different routes for viral injection administration were used. In the formal experiment, the footpad inoculation model was developed to investigate the arthritis associated with CHIKV infection, whereas intraperitoneal injection (i.p.) was adopted in the preliminary study. Second, the viral infectious titer may not have been appropriate for the footpad inoculation model, as early death occurred within 2 days after infection with CHIKV in the isotype control group. Furthermore, for better therapeutic treatment, rational dosing of Nbs should be optimized in future studies. Notably, the optimal timing of Nb treatment is also crucial for antibody efficacy. As previously reported, different time points such as 6 h, 12 h, 24 h, and 36 h could be set up to evaluate the time course and treatment effects of therapeutic antibodies. Further studies are needed to explore the hidden epitopes targeted by Nbs and clarify the critical factors affecting the protective efficacy of Nbs, which provides a new direction for the development of CHIKV-specific protective antibodies.

## 4. Materials and Methods

### 4.1. Cells and Viruses

The human embryonic kidney cell line (HEK293T, ATCC, Manassas, VA, USA, Cat# CRL-11268), baby hamster kidney fibroblast cell line (BHK-21, ATCC, Cat# CRL-8544), and African green monkey kidney cells (Vero E6, ATCC, Cat# CRL-1586) were cultured at 37 °C and 5% CO_2_ in Dulbecco’s Modified Eagle’s Medium (DMEM) (Gibco, Grand Island, NE, USA, Cat# C11995500BT) supplemented with 10% fetal bovine serum (FBS), 100 μg/mL streptomycin, and 100 IU/mL penicillin (Gibco, Cat# 15140122). Expi293F human cells (Thermo Fisher Scientific, Waltham, MA, USA, Cat# A14527), derived from the 293F cell line, were cultivated in Expi293 Expression Medium at 37°C in a humidified 8% CO2 shaker rotating at 125 rpm. Expisf9 insect cells (Thermo Fisher Scientific, Waltham, MA, USA, Cat# A35243) were cultured at 27 °C in a shaker rotating at 120 rpm using Gibco™ Sf-900™ III SFM (Gibco, Grand Island, NE, USA, Cat# 12658027).

A live-attenuated strain of CHIKV [40] was propagated in BHK-21 cells grown in RPMI 1640 medium supplemented with 2% FBS at 37 °C.

### 4.2. Alpaca Immunization

To isolate neutralizing Nbs against CHIKV, an alpaca was subcutaneously immunized with 1 mg of the recombinant CHIKV p62-E1 heterodimer in the presence of Freund’s adjuvant on day 0, and booster immunizations with 0.5 mg of p62-E1 protein in the presence of Freund’s adjuvant were administered on days 21, 42, 63, and 84. Whole blood was collected one week after the last immunization for VHH phage display library construction. Total RNA was extracted from peripheral blood mononuclear cells (PBMCs) and used as a template for first-strand cDNA synthesis with an oligo dT primer. Subsequently, VHH-encoding sequences were amplified from the cDNA and cloned into the phagemid vector pComb3X, and then HA and His6 tags (AAAYPYDVPDYGSHHHHHH) were added at the C-terminus. Electrocompetent *E. coli* XL1-Blue cells were transformed with the recombinant plasmids, resulting in a VHH library of approximately 4.9 × 10^8^ independent transformants. The resulting XL1-Blue library stock was then infected with VCSM13 helper phages to generate a VHH-presenting phage library [41].

### 4.3. VHH Library Genenration

Phages displaying VHHs specific for CHIKV were enriched after two rounds of biopanning on 20 µg of immobilized CHIKV p62-E1 proteins in one well of a microwell plate (type II, F96 Maxisorp, Nunc). For each panning round, an uncoated well was used as a negative control. The wells were washed five times with phosphate-buffered saline (PBS) containing 0.1% Tween 20 and blocked with 5% milk powder in PBS for 1 h at 37 °C. Approximately 5 × 10^12^ phages were added to the coated well and incubated for 2 h at room temperature. Non-specifically bound phages were removed by washing with PBS containing 0.1% Tween 20 (10 times in the first panning round and 15 times in the second panning round). The retained phages were eluted with glycine-HCl and subsequently neutralized with 1 M Tris-HCl (pH 7.4). The collected phages were amplified in *E. coli* XL1-Blue cells, infected with VCS M13 helper phages, and subsequently purified using PEG 8000/NaCl precipitation for the next round of selection. Enrichment after each panning round was determined by infecting XL1-Blue cells with 10-fold serial dilutions of the collected phages.

After panning, individual clones were picked to inoculate 2 × YT (yeast extract tryptone) medium supplemented with 100 μg/mL ampicillin overnight at 37 °C. The cell-free phage supernatant was detected by phage ELISA with HRP-conjugated goat anti-M13 IgG antibody (Sino Biological, Beijing, China). OD_450_ values ≥ 0.5 and a P (positive OD_450_)/N (negative OD_450_) greater than 3 P/N ratios were determined as positive clones. Positive candidates were sequenced (Sangon Biotech, Shanghai, China) and aligned. Five percent bovine serum albumin (BSA) was used as a negative control for each round.

### 4.4. Protein Purification

Sequences encoding VHHs were amplified from the sorted phage clones and inserted into pcDNA3.4 expression vectors to express VHH in conjunction with a human IgG1 Fc fragment. Specifically, VHH genes were cloned into the multiple cloning sites of pCDNA3.4 containing the upstream CMV promoter, tPA signal peptide, and the downstream human IgG1 Fc gene fragment and SV40 poly (A) signal sequence. Expression and production of Nbs were conducted by transfecting the expression vectors into the Expi293F cells using an ExpiFectamine 293 Transfection Kit (Thermo Fisher Scientific, Cat# A14526). After 4 days of continuous culture, the supernatants were harvested by centrifugation and subsequently filtered through 0.22 μm syringe filters (PALL, Bristol, UK, Cat# 4612) or disposable vacuum systems (Biosharp, Beijing, China, Cat# BS-500-XT). Recombinant proteins were purified on an ÄKTA Pure 150 purification system (GE Healthcare, Piscataway, NJ, USA) using a 5 mL Protein A column (binding buffer: PBS, pH 7.5; elution buffer: 0.1 M Glycine, pH 2.7).

All proteins were analyzed for purity by SDS–PAGE, and the concentration was determined using a BCA protein assay kit (Merck Millipore, Darmstadt, Germany, Cat# 23225) or the UV 280 nm absorption method.

### 4.5. Enzyme-Linked Immunosorbent Assay (ELISA)

Purified p62-E1 proteins (2 μg/mL) were immobilized on ELISA 96-well plates overnight in a sodium bicarbonate buffer (50 mM sodium carbonate, 50 mM sodium bicarbonate, pH 9.6) at 4 °C. Plates were washed with PBST (PBS + 0.02% Tween 20) and blocked with blocking buffer (PBS + 2% BSA) for 1 h at 37 °C. Blocking buffer was removed and plates were washed with PBST (PBS + 0.02% Tween 20). Purified antibodies were diluted with antibody diluent buffer (PBS + 0.2% BSA + 0.01% Tween 20) in 3-fold dilutions, starting at 1 μg/mL. Then, 100 μL of the diluted antibodies was added to each well (100 μL/well) and incubated for 1 h at 37 °C. Plates were washed with PBST (PBS + 0.02% Tween 20) and incubated with an HRP-conjugated goat anti-human IgG Fc antibody (Abcam97225, Cambridge, UK, 1:10,000 dilution) for 1 h at 37 °C. Plates were washed again. Enhanced TMB single-component substrate solution (Solarbio, Beijing, China, Cat# PR1200) was added to each well of the plate (100 μL/well) and incubated in the dark at room temperature for 5 min. The reaction was stopped with the addition of 2 M H_2_SO_4_ (50 μL/well), and the absorbance values were determined at 450 nm with a reference wavelength of 630 nm. Binding curves were generated by performing a 4-parameter non-linear regression dose–response analysis, and the half-maximal effective concentration (EC_50_) values of each Nb were calculated using GraphPad Prism V8.

### 4.6. Virus Neutralization Assay

The day before infection, BHK21 cells were diluted to 1.5 × 10^5^ cells/mL in medium (DMEM + 10% FBS) and seeded into 96-well cell culture plates with a volume of 200 μL/well and cultured at 37 °C in a 5% CO_2_ cell incubator. On the day of transfection, Nbs were diluted with medium (DMEM + 2% FBS) to the initial concentration (100 μg/mL), then 4-fold serially diluted. Then, 100 μL virus and 100 μL Nbs were fully mixed and incubated at 37 °C in a 5% CO_2_ cell incubator for 1 h. After incubation, cell culture supernatants were removed and 200 μL of the incubated virus–antibody mixtures was added to each well and cultured at 37 °C in a 5% CO_2_ cell incubator. After 72 h, the cell culture supernatant was discarded, and 50 ul/well of crystal violet stain with 4% paraformaldehyde was added and incubated for 30 min at room temperature for cell fixation and staining. Then, the staining solution was discarded and washed with water 5 times, and 100 μL/well decolorization solution (50 mL of absolute ethanol, 0.1 mL of acetic acid, water to 100 mL) was added for full dissolution. Using a microplate reader to measure the OD_570_ value with a reference wavelength of OD_630_, the cell survival rate and antibody concentration were fitted with GraphPad Prism 8 to calculate the half-maximal inhibitory concentration (IC_50_) value.

### 4.7. Surface Plasmon Resonance (SPR)

SPR measurements were performed using a Biacore T200 instrument (Cytiva, Shanghai, China) to measure the kinetics and affinity of anti-CHIKV antibodies binding to CHIKV p62-E1. Experiments were performed at a flow rate of 30 μL/min and a temperature of 25 °C using HBS-EP (0.01 M HEPES pH 7.4, 0.15 M NaCl, 3 mM EDTA, 0.005% *v*/*v* Surfactant P20) as running buffer. First, the antibody was diluted to 1 μg/mL and captured by the Protein A chip at a flow rate of 10 μL/min. Then, the purified CHIKV p62-E1 protein was injected at concentrations of 25 nM, 12.5 nM, 6.25 nM, 3.125 nM, 1.5625 nM, and 0 nM, passed through the Protein A chip that had captured the antibody at a flow rate of 30 μL/min, bound for 120 s to determine the binding rate (Ka), and then dissociated for 600 s to determine the dissociation rate (Kd). The chip surface was regenerated between each cycle using 10 mM glycine, pH 1.5. Sensorgrams for the association and dissociation phases were fitted to a 1:1 binding model using Biacore Insight Evaluation software (Cytiva, Shanghai, China). Finally, the affinity of the targeted antibody was calculated by dividing Kd by Ka.

### 4.8. Competition-Binding ELISA

First, 200 μg of antibodies was incubated with a 20-fold molar excess of biotin at room temperature for 1 h. Biotinylated antibodies were buffer-exchanged several times to remove excess biotin, and the concentration was determined using NanoVue Plus (GE Healthcare) at 280 nm.

The competition-binding ELISA was performed in 96-well plates. Purified CHIKV p62-E1 proteins (2 μg/mL) were immobilized on ELISA plates overnight in a sodium bicarbonate buffer (50 mM sodium carbonate, 50 mM sodium bicarbonate, pH 9.6) at 4 °C. Plates were washed with PBST (PBS + 0.02% Tween 20) and blocked with blocking buffer (PBS + 2% BSA) for 1 h at 37 °C. Blocking buffer was removed and plates were washed with PBST (PBS + 0.02% Tween 20). The first antibody (20 μg/mL) in antibody diluent buffer (PBS + 0.2% BSA+ 0.01% Tween 20) was added at 50 μL/well and incubated for 30 min at 37 °C. The biotinylated second antibody (2 μg/mL; final concentration of 1 μg/mL) in antibody diluent buffer (PBS + 0.2% BSA + 0.01% Tween 20) was added at 50 μL/well and incubated for 1 h at 37 °C. Plates were washed with PBST (PBS + 0.02% Tween 20) and incubated with an HRP-conjugated streptavidin (Abcam, Cat# AB7403) for 1 h at 37 °C. Plates were washed again. Enhanced TMB single-component substrate solution (Solarbio, Cat# PR1200) was added to each well of the plate (100 μL/well) and incubated in the dark at room temperature for 5 min. The reaction was stopped with the addition of 2 M H_2_SO_4_ (50 μL/well), and the plates were read at 450 nm. The percentage of bound biotinylated antibodies was calculated by comparing the absorbance value in the presence of competitors to that in the presence of an irrelevant control Nb, RVFV-NA137-Fc. (The nanobody against RVFV was screened by our team, and the data are unpublished.) Antibodies were considered to be competing for the same or close epitope if the percentage of bound detected Nb was <33%. Antibodies were assumed to bind to different sites if the percentage value was >66%. A group of intermediate competitive antibodies was identified if their percentage value was in the range of 33–66% [42].

### 4.9. In Vivo Animal Challenge Experiment

For the prophylactic study, 4–6-week-old female/male Type 1 interferon receptor knockout mice (*Ifnar^−/^^−^*) [23] (8 animals in each group) were treated via intraperitoneal injection (i.p.) with 100 μg of the indicated Nbs and isotype antibody RVFV-NA137-Fc 24 h post-treatment. Mice were subjected to subcutaneous inoculation in the left rear footpad with 1 × 10^3^ TCID_50_ of the CHIKV live-attenuated strain. The mice were observed daily for mortality, morbidity, body weight, and ipsilateral/contralateral foot swelling for up to 14 days after infection. Three animals from each group were euthanized by cervical dislocation on day 3 after infection to determine infectious virus in the heart, liver, spleen, lung, kidney, brain, muscle, and joints by RT-qPCR. The tissues were fixed with 10% formalin for 48 h, embedded in paraffin, and sectioned. Next, the fixed tissue sections were subjected to hematoxylin and eosin (H&E) staining.

For the therapeutic study, 4–6-week-old female/male *Ifnar^−/^^−^* mice (8 animals in each group) were subjected to subcutaneous inoculation in the left rear footpad with 1 × 10^3^ TCID_50_ of the CHIKV live-attenuated strain. Mice were treated via intraperitoneal injection (i.p.) with 100 μg of the indicated Nbs and isotype antibody RVFV-NA137-Fc 24 h post-exposure. The mice were observed daily for mortality, morbidity, body weight, and ipsilateral/contralateral foot swelling for up to 14 days after infection. Three animals from each group were euthanized by cervical dislocation on day 3 after infection to determine infectious virus in the heart, liver, spleen, lung, kidney, brain, muscle, and joints by RT-qPCR. The tissues were fixed with 10% formalin for 48 h, embedded in paraffin, and sectioned. Next, the fixed tissue sections were subjected to hematoxylin and eosin (H&E) staining.

### 4.10. Viral Load Determination by RT-qPCR

Viral burden was determined using RT-qPCR using RNA isolated from viral stocks as a standard to determine viral RNA copies. Briefly, RNA was isolated from tissues using the RNeasy Mini Kit (Qiagen, Dusseldorf, North Rhine-Westphalia, Germany, Cat# 74104) according to the manufacturer’s protocol. Reverse transcription of cDNA was performed using HiScript III RT SuperMix (Vazyme, Nanjing, China, Cat# R323), and RT-qPCR was performed using 2 × Taq Pro Universal SYBR qPCR Master Mix (Vazyme, Cat# Q712) with the following CHIKV primer pair:

CHIKV9756-9777-F, 5′-AGCTACCGTCCCTTTCCTGCTTA-3′ and

CHIKV 9843-9866-R, 5′-CAAAACAAAGGTTGCTGCTCGTT-3′.

### 4.11. Construction of Cell–Cell Fusion Models and Cell–Cell Fusion Inhibition

To construct a visible cell fusion system, the gene encoding the E protein from CHIKV was cloned into the pIRES2-EGFP vector [17]. A total of 1 × 10^6^ HEK293T cells were seeded into 6-well culture plates in 2 mL of DMEM supplemented with 10% FBS and incubated at 37 °C and 5% CO2 for 16 h. The pIRES2-CHIKV E-EGFP and empty pIRES2-EGFP vector (control) (4 µg) plasmids were transfected into HEK293T cells using the Turbofect transfection reagent (8 µL) (ThermoFisherScientific, Cat# R0532). At 48 h post-transfection, the HEK293T effector cells expressing the CHIKV E protein (2.5 × 10^5^ cells per well) were co-incubated with Vero E6 target cells (2.5 × 10^5^ cells per well) in a 24-well glass-bottom plate pre-coated with type I collagen (BD Bioscience, Beijing, China, Cat#354236) at 37 °C for 24 h. Subsequently, 200 µL/well of medium with or without each antibody (final concentration, 10 μg/mL) was added. The cells were then incubated at 37 °C for 1 h, and the culture medium was replaced with the neutral-pH (pH 7.0) or low-pH (pH 5.5) medium. The cells were incubated for an additional 2 h at 37 °C, and the cell monolayers were fixed and stained. Cell fusion images were captured using fluorescence microscopy (Biotek), and the number of syncretic cells within at least 5 randomly selected fields was counted. The fused cells were much larger and had weaker fluorescence intensity than the unfused cells due to the diffusion of EGFP from one cell to multiple cells. The fusion rate = (fused cell number)/(fused cell number + unfused cell number) ×100%.

### 4.12. Egress Inhibition Assay

Vero cells in 96-well plates were inoculated with CHIKV. After 1 h at 37 °C, cells were washed 6 times with medium. The cells were incubated with 200 µL of medium containing dilutions of the indicated Nbs at 37 °C. Supernatant was harvested at 1 h and 6 h after infection, and the viral RNA was extracted using a QIAamp Viral RNA Mini Kit (Qiagen, Cat# 52904) following the manufacturer’s protocol. Isolated RNA was quantified with a Taq Pro Universal SYBR qPCR Master Mix (Vazyme, Cat# Q712) on a QuantStudio 3 (Thermo Fisher Scientific) using the following CHIKV primer pair:

CHIKV9756-9777-F, 5′-AGCTACCGTCCCTTTCCTGCTTA-3′ and

CHIKV9843-9866-R, 5′-CAAAACAAAGGTTGCTGCTCGTT-3′.

### 4.13. Mxra8 Blocking Experiment

The ELISA was performed in 96-well plates. Purified CHIKV p62-E1 proteins (2 μg/mL) were immobilized on ELISA plates overnight in a sodium bicarbonate buffer (50 mM sodium carbonate, 50 mM sodium bicarbonate, pH 9.6) at 4 °C. Plates were washed with PBST (PBS + 0.02% Tween 20) and blocked with blocking buffer (PBS + 2% BSA) for 1 h at 37 °C. Blocking buffer was removed and plates were washed with PBST (PBS+0.02% Tween 20). CHIKV Nbs (N033-Fc and N053-Fc) or Mxra8 human-Fc fusion protein (all at 10 µg/mL) were included with the immobilized proteins on the plates for 30 min. Subsequently, without washing, serially diluted Mxra8 human-Fc-Biotin fusion protein was added for 1 h at 37 °C. Plates were washed with PBST (PBS + 0.02% Tween 20) and incubated with HRP-conjugated streptavidin (Abcam, Cat# AB7403) for 1 h at 37 °C. Plates were washed again. Enhanced TMB single-component substrate solution (Solarbio, Cat# PR1200) was added to each well of the plate (100 μL/well) and incubated in the dark at room temperature for 5 min. The reaction was stopped with the addition of 2 M H_2_SO_4_ (50 μL/well), and the plates were read at 450 nm [24].

### 4.14. Quantification and Statistical Analysis

All statistical analyses were performed using GraphPad Prism 8.0.1, and the significance was defined as ns, *p* ≥ 0.05; *, *p* < 0.05; **, *p* < 0.01; ***, *p* < 0.001; ****, *p* < 0.0001. Details of the statistical tests are included in the figure legends.

## Data Availability

The original contributions presented in the study are included in the article and Supplementary Materials, and further inquiries can be directed to the corresponding authors.

## Supplementary Materials

The following supporting information can be downloaded at https://www.mdpi.com/article/10.3390/ijms26093982/s1.
