# Supplementary material for: Highly Potent Neutralizing Nanobodies Acting Against Chikungunya Virus Infection via Inhibiting Multiple Stages of the Viral Life Cycle
Source: Int J Mol Sci. 2025 Apr 23;26(9):3982. doi: 10.3390/ijms26093982 (PMC12071869; doi:10.3390/ijms26093982)
Supplement: Supplementary file 1 [file ijms-26-03982-s001.zip › ijms-3548703-supplementary.pdf]

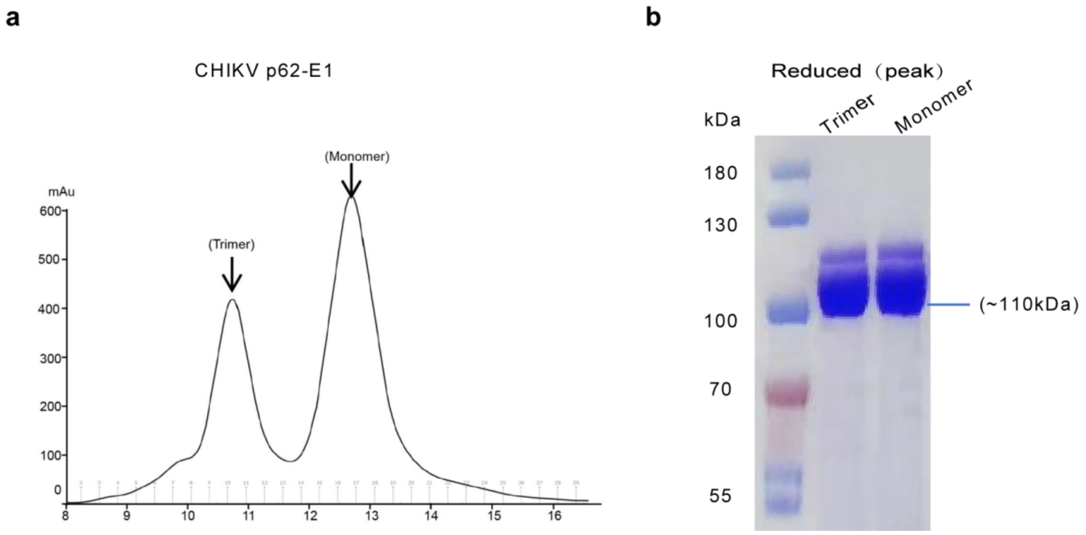

Supplementary Figure S1. The isolation of the trimeric and monomeric forms of CHIKV p62-E1. (a) The trimeric and monomeric forms of CHIKV p62-E1 were separated by molecular exclusion chromatography. (The first peak represents the trimer, and the second peak represents the monomer.). (b) The purity of CHIKV p62-E1 was assessed by SDS-PAGE.

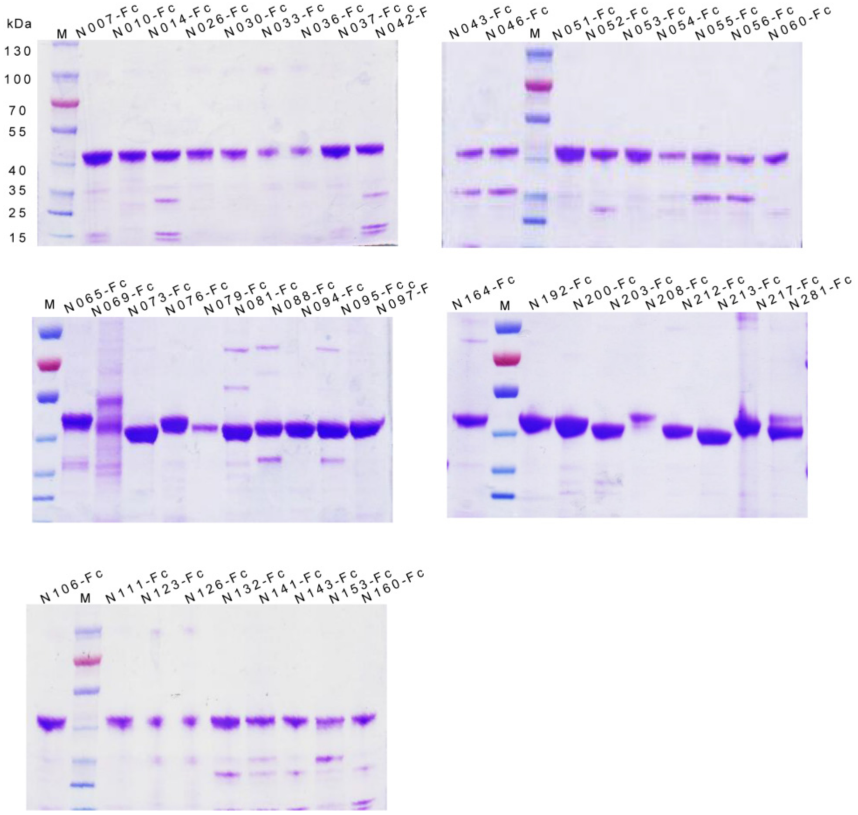

Supplementary Figure S2. The expression of CHIKV-specific Nbs. The purity of

CHIKV-specific Nbs was assessed by SDS-PAGE.

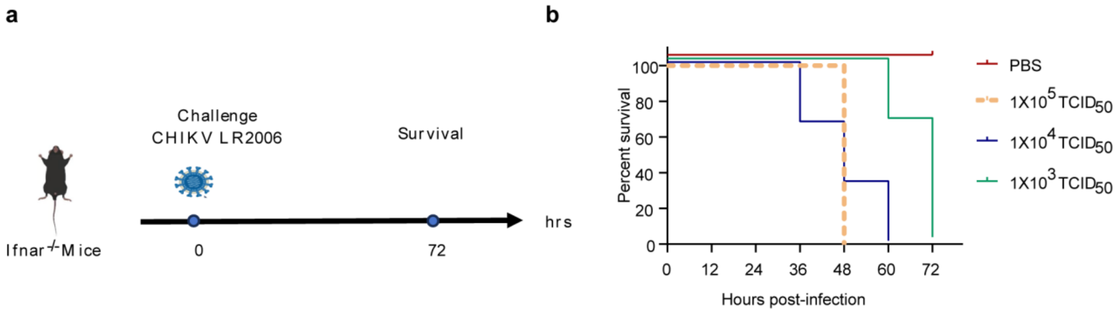

Supplementary Figure S3. Establishment of a lethal model of mouse CHIKV challenge. (a) Experimental schedule for CHIKV challenge. (b) Survival curves of mice at different challenge doses. (Survival curve analysis was performed using the log-rank test.)

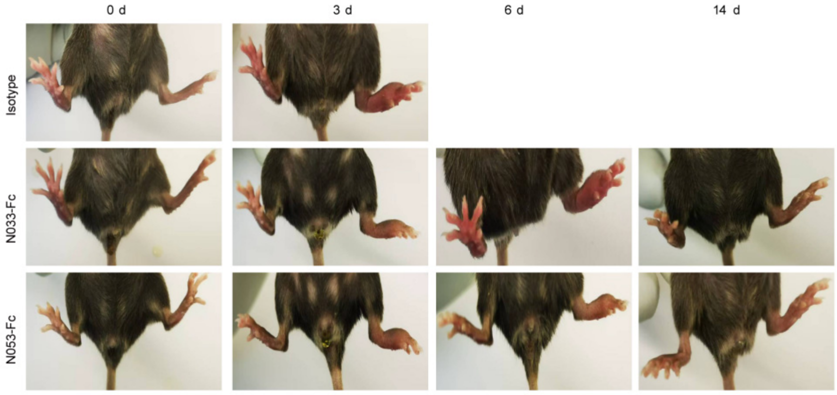

Supplementary Figure S4. Representative images of mice foot swelling at 0, 3, 6, 14 days in the prophylaxis test. At 6 and 14 days, all isotype control mice died, and no images were captured. The image shows foot swelling, N033-Fc and N053-Fc effectively alleviated the symptoms to nearly baseline levels by 14 dpi.

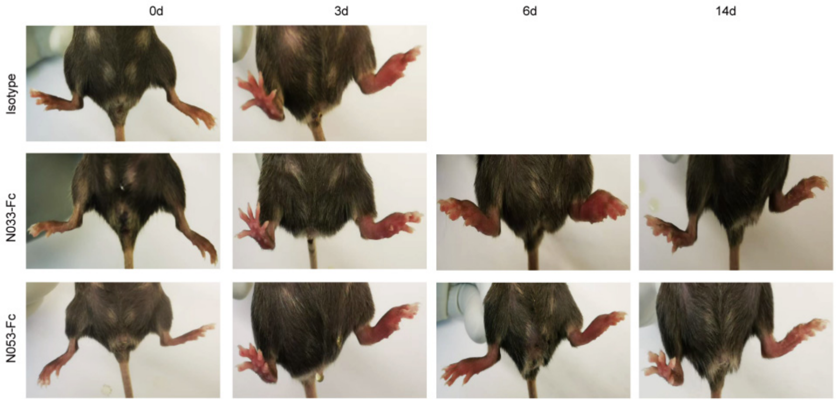

Supplementary Figure S5. Representative images of mice foot swelling at 0, 3, 6, 14 days in the therapy test. At 6 and 14 days, all isotype control mice died, and no images were captured. The image shows foot swelling, N033-Fc and N053-Fc effectively alleviated the symptoms to nearly baseline levels by 14 dpi.
